# Comparisons of Antibody Populations in Different Pre-Fusion F VLP-Immunized Cotton Rat Dams and Their Offspring

**DOI:** 10.3390/vaccines8010133

**Published:** 2020-03-18

**Authors:** Lori M. Cullen, Marina S. Boukhvalova, Jorge C. G. Blanco, Trudy G. Morrison

**Affiliations:** 1 Department of Microbiology and Physiological Systems, University of Massachusetts Medical School, Worcester, MA 01655, USA; Lori.McGinnes@umassmed.edu; 2 Sigmovir Biosystems Inc., Rockville, MD 20850, USA; m.boukhvalova@sigmovir.com; 3 Program in Microbiology and Immunology, University of Massachusetts Medical School, Worcester, MA 01655, USA

**Keywords:** vaccines, virus-like particles, pre-fusion F proteins, antibodies, maternal antibodies

## Abstract

Respiratory syncytial virus (RSV) infection poses a significant risk for infants. Since the direct vaccination of infants is problematic, maternal vaccination may provide a safer, more effective approach to their protection. In the cotton rat (CR) model, we have compared the immunization of pregnant CR dams with virus-like particles assembled with the prototype mutation stabilized pre-fusion F protein, DS-Cav1, as well two alternative mutation stabilized pre-fusion proteins (UC-2 F, UC-3 F) and showed that the alternative pre-fusion F VLPs protected the offspring of immunized dams significantly better than DS-Cav1 F VLPs (Blanco, et al. J. Virol. 93: e00914). Here, we have addressed the reasons for this increased protection by characterizing the specificities of antibodies in the sera of both immunized dams and their offspring. The approach was to measure the levels of total anti-pre-F IgG serum antibodies that would block the binding of representative pre-fusion specific monoclonal antibodies to soluble pre-fusion F protein targets. Strikingly, we found that the sera in most offspring of DS-Cav1 F VLP-immunized dams had no mAb D25-blocking antibodies, although their dams had robust levels. In contrast, all offspring of UC-3 F VLP-immunized dams had robust levels of these D25-blocking antibodies. Both sets of pup sera had significant levels of mAb AM14-blocking antibodies, indicating that all pups received maternal antibodies. A lack of mAb D25-blocking antibodies in the offspring of DS-Cav1 F VLP-immunized dams may account for the lower protection of their pups from challenge compared to the offspring of UC-3 F VLP-immunized dams.

## 1. Introduction

Respiratory syncytial virus (RSV) is the most common lower respiratory tract viral pathogen of neonates and infants [1]. This virus accounts for 33.1 million acute lower respiratory tract infections, 3.2 million hospitalizations and an estimated yearly mortality of 118,200 for this population [2]. RSV infections are a common cause of infant physician office visits [3]. Despite decades of effort, no vaccine has yet been licensed. Furthermore, the use of any licensed RSV vaccine for immunization of infants will be problematic due to safety issues and the immaturity of their immune systems. As a result of these difficulties, maternal immunization for the protection of their offspring is considered a better approach [4,5,6,7,8,9,10,11,12,13,14].

We have developed novel virus-like particle (VLP) vaccine candidates for RSV [14,15,16,17,18]. In contrast to soluble proteins, VLPs robustly stimulate immune responses without the complications of adjuvant addition [19]. VLPs are safer as vaccines for many populations, such as the very young, compared to infectious, attenuated, or vector viruses, since they do not contain a genome and do not produce a spreading infection. Our VLPs are based on the core proteins of Newcastle disease virus (NDV), NP and M proteins, and they are assembled with the RSV F and G protein ectodomains fused to the transmembane and cytoplasmic domains of the NDV F and HN proteins, respectively.

There has been a resurgence of interest and activity in RSV vaccine development due to the ground-breaking studies of McLellan, et al. who succeeded in solving the crystal structure of the RSV pre-fusion F protein and identifying a set of mutations in the F protein, termed DS-Cav1, which stabilized the pre-fusion form of the F protein [20,21]. We have reported that VLPs assembled with the DS-Cav1 mutant F protein stimulate, in mice and in cotton rats, neutralizing antibody titers much higher than those induced by VLPs assembled with the post-fusion F protein or wild-type F protein [16,22]. Furthermore, the immunization of cotton rat dams with DS-Cav1 F VLPs protected their offspring from RSV challenge [14].

Since the description of DS-Cav1 F protein, a number of other laboratories and companies have identified different sets of mutations that reportedly stabilize the pre-fusion F protein [23,24,25,26,27,28,29]. A very important question for vaccine development is whether the different mutation-stabilized pre-fusion F proteins are indeed the same in terms of structure, antibodies induced, and protection from RSV challenge afforded by their use as immunogens. We have recently addressed these questions by comparing the reactivity to monoclonal antibodies and the immunological properties of virus-like particles (VLPs) assembled with different versions of mutation-stabilized pre-fusion F proteins [18,30]. We have reported that five different pre-fusion F proteins, in VLPs, bind differently to representative pre-fusion specific monoclonal antibodies (mAb) [18]. Compared to VLPs assembled with DS-Cav1 F protein, two of these alternative mutation-stabilized pre-fusion F protein VLPs induced, in mice, neutralization titers 3 to 4-fold higher than DS-Cav1 F VLPs [18]. Furthermore, we showed that the specificities of the population of antibodies induced in mice by the five different VLP-associated pre-fusion F proteins were different as defined by differences in the serum inhibition of binding of representative monoclonal antibodies to the soluble forms of the pre-fusion F protein [18]. These combined results indicate that not all mutant stabilized pre-fusion F proteins are the same with respect to the population of antibodies they induce.

Using cotton rats (CR), the preferred animal model for RSV [7,14,31,32], we reported that the immunization of pregnant animals with one of these alternative pre-fusion F VLPs increased their serum-neutralizing antibody (NAb) titers and significantly increased the protection from RSV challenge of their offspring compared to the immunization of dams with DS-Cav1 F VLPs [30]. These results raise the question of why there are differential levels of protection of offspring upon immunization of their dams with the different VLP-associated pre-fusion F proteins. To begin to account for these differences, we have compared the specificities of the antibodies induced by the different VLPs in CR dams and the specificities of antibodies transferred to their offspring. We report that DS-Cav1 F VLPs and other pre-fusion F VLPs induced different populations of antibodies in pregnant CR dams Most importantly, the specificities of the population of anti-F protein antibodies in the serum of offspring did not, in some cases, correlate with the specificities of the population of antibodies in their dams. The results point to the importance of the selection of the appropriate mutant stabilized pre-fusion F to formulate a maternal vaccine for the optimal protection of neonates.

## 2. Materials and Methods

### 2.1. Cells, Plasmids, Viruses

ELL-0, used for VLP preparation, and Vero cells, used for plaque assays, were obtained from the American Type Culture Collection and grown in DMEM (Invitrogen, ThermoFisher Scientific; Waltham, MA, USA) supplemented with penicillin, streptomycin (Invitrogen), and 5% (Vero cells) or 10% fetal bovine serum (ELL-0) (Invitrogen). Expi293F cells, used for soluble protein production, were obtained from ThermoFisher/Invitrogen and grown in Expi293 media (ThermoFisher/Gibco/Invitrogen). RSV, prototype long strain (ATCC VR-26), was used for infection and challenge.

VLPs contained the RSV F and G protein ectodomains (derived from RSV, strain A2) and were assembled with the Newcastle disease virus (NDV) core proteins NP and M, as previously described [15,18,22,33]. The F proteins were assembled into VLPs as chimera proteins with the RSV F protein ectodomain fused to the NDV F transmembrane (TM) and cytoplasmic tail (CT). The G protein was assembled as a chimera protein with the RSV G protein ectodomain fused to the NDV HN, TM, and CT domains [34]. The construction, expression, and incorporation of the chimera proteins into VLPs have been previously described. Four VLPs were assembled, each with a different mutant F protein ectodomain: DS-Cav1 F protein, post F protein, UC-2 F protein, and UC-3 F protein. Mutations in DS-Cav1 protein have been previously described [21]. UC-2 F and UC-3 F proteins both had deletions of the p27 sequence, including the two cleavage sites combined with the insertion of a linker sequence GSGSGRS. In addition, UC-2 F had two amino acid substitutions (N67I, S215P), and UC-3 F had three (N67I, S215P, D486N) [18]. These mutants are similar to SC-DM and SC-TM (single chain double mutant and single chain triple mutant, respectively), as described by Krarup et al. [27].

The constructions of the genes encoding the soluble DS-Cav1 F protein and soluble G protein have been previously described [18,22]. The soluble UC-3 F protein was constructed similarly to the soluble DS-Cav1 protein, as previously described [18].

### 2.2. Antibodies

Human mAb D25, mAb AM14, and palivizumab used for antibody-blocking experiments were the generous gifts of Jason McLellan and Dr. Jorge Blanco. Secondary antibody against human IgG was purchased from Southern Biotech.

### 2.3. VLP Preparation, Purification, and Characterization

The preparation of VLPs used as immunogens (abbreviated as DS-Cav1 F VLPs, post-F VLPs, UC-2 F VLPs, UC-3 F VLPs) has been previously described [18]. Briefly, VLPs were harvested from ELL-0 cells transfected with cDNAs encoding the NDV NP and M protein, the chimera protein H/G, and one of the three pre-F proteins or the post-F protein. VLPs were collected from cell supernatants and purified by sequential pelleting and sucrose gradient fractionation as previously described. The conformation of different F proteins in the VLP preparations was verified by reactivity to mAb, as has been previously published [14,18].

### 2.4. Preparation of Soluble F Proteins

Soluble F or G proteins were prepared in Expi293F cells. The cells were transfected with cDNAs encoding the soluble DS-Cav1 pre-F protein, the soluble UC-3 pre-F protein, or the soluble G protein. At six days post-transfection, total cell supernatants were collected, and cell debris was removed by centrifugation. Then, soluble polypeptides were purified on columns using the His tag and the strep tag and validated as previously described [14].

### 2.5. Quantification of NP, M, H/G, and VLP Associated F Proteins or Soluble F Proteins

Quantifications of NP, M, RSV G protein, and RSV F proteins in VLPs or in soluble F or G protein preparations were accomplished after their separation in polyacrylamide gels followed by silver staining (Pierce Silver Stain, ThermoFisher) or Western blots of the proteins in parallel with protein standards, as previously described [35].

### 2.6. Animal Studies

Animal studies have been described previously [30]. Briefly, *Sigmodon hispidus* cotton rats were obtained from an inbred colony maintained at Sigmovir Biosystems, Inc. (Rockville, MD USA). Three-week-old female cotton rats (4–5/group) were bled by retro-orbital plexus puncture and then primed by RSV A/Long infection intranasally using a dose of 10^5^ PFU/animal in 50 μl. After 56 days (8 weeks), females were set up in breeding pairs with RSV-negative males. At day 70 (2 weeks into gestation), different groups of pregnant cotton rats were immunized with DS-Cav1, Post-F, UC-2 F VLPs, or UC-3 F VLPs with 100 μg total VLP protein/animal (20 μg F protein), or TNE buffer (50 mM Tris-HCl, pH 7.4, 150 mM NaCl, 5 mM EDTA). Females were bled for serum collection at day 84 (just before delivery). Dams delivered pups at approximately day 84. All pups were eye-bled and challenged with RSV A/Long (10^5^ PFU/animal) at 4 weeks of age. On day 4 post-infection, all pups were sacrificed for nose and lung viral titers. All studies were conducted under applicable laws and guideline and after approval from the Sigmovir Biosystems, Inc. Institutional Animal Care and Use Committee.

### 2.7. Blocking of mAb Binding to Soluble DS-Cav1 F or UC-3 F Proteins

**Figure A1:**
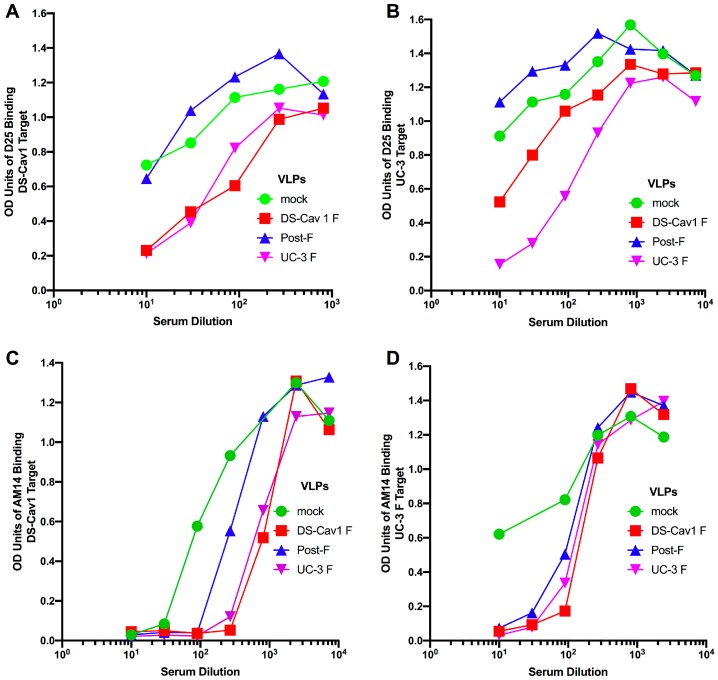
Titration of binding of mAb D25 and AM14 to soluble pre-F targets in presence of different dilutions of CR dam sera. Shown are illustrations of the determinations of dilution of sera induced by different VLPs that block 50% of the binding of D25 to soluble DS-Cav1 F (panel A) or UC-3 target (panel B) or that block the binding of mAb AM14 to soluble DS-Cav1 F protein target (panel C) or to soluble UC-3 F target (panel D). Using the total ng/mL of anti-pre-F binding IgG in each serum, the ng/mL of anti-pre-F IgG in the dilution of sera that blocks 50% of the binding of the mAb can be calculated. Total ng/mL of pre-F binding IgG in the dam sera were: Mock, 2.3 × 10^5^; DS-Cav1 F VLP sera, 4.0 × 10^6^; post F VLP sera, 5.0 × 10^6^; UC-3 F VLP sera, 5.5 × 10^6^. Total ng/mL of anti-pre-F IgG in pup sera was 3 × 10^5^ ng/mL. All titrations were done two or three times.

To measure the abilities of polyclonal sera to block the binding of mAbs to the F protein target, different dilutions of sera, in PBS-1% BSA (phosphate buffered saline containing 1% bovine serum albumin), were incubated for 1 h at room temperature in wells of Ni-coated microtiter plates (Pierce/ThermoFisher) containing 50 ng of pre-bound soluble DS-Cav1 pre-F protein or soluble UC-3 pre-F protein. Ni-coated plates were used in order to bind the soluble pre-F proteins via the histidine tag at the carboxyl terminus of the protein and thus orienting the protein in the well with the apex of the molecule projecting upwards as in virus particles. After removal of the serum, the wells were incubated with 200 ng/mL of one of the purified mAb and diluted in PBS-1% BSA for 10 min at room temperature. Then, the mAb was removed, the plate was washed in PBS, and it was incubated with goat anti-human IgG coupled to HRP (horse radish peroxidase). After incubation for 1 h at room temperature, the bound HRP was detected as in ELISA assays. The total anti-pre-F IgG in the different serum dilutions used for mAb blocking was determined using a standard curve of purified CR IgG in order to measure the ng of serum anti-pre-F antibody in the dilution that blocked the binding of the mAb (illustrated in Figure A1).

### 2.8. Statistical Analysis

Statistical analyses (student T test) of data were accomplished using Graph Pad Prism 7 software.

## 3. Results

### 3.1. Specificities of Anti-Pre-Fusion F Protein Antibodies Defined Using Soluble DS-Cav1 F Targets

We have previously described and compared immune responses in CR immunized with DS-Cav1 F VLPs and two alternative pre-fusion F VLPs [30]. CR females were RSV primed by intranasal infection, mated at 56 days after the RSV prime, and then immunized at various times of gestation with DS-Cav1 F, UC-2 F, UC-3 F, post-F VLPs, or mock-immunized. Sera were collected at day 84, 4 weeks of gestation, just before the delivery of offspring. Sera were harvested from the offspring at 4 weeks post-birth. The total ng/mL of anti-pre-F IgG in dams was shown to be similar in all VLP-immunized animals and 10-fold higher than in the mock-immunized animals [30]. Levels (ng/mL) of total anti-pre-F IgG in all pup sera were also very similar and approximately 10-fold lower than titers in their dams [30]. For the experiments reported here, comparing the specificities of the antibodies induced by the three different pre-fusion F VLPs, we have utilized sera from selected groups of animals from this previously reported study. Specifically, sera from animals immunized at two weeks of gestation and offspring from these dams were selected for analysis in this study.

We have previously compared the relative specificities of murine antibodies induced by different VLPs measuring the concentration of anti-pre-F binding IgG in sera that will block the binding of pre-fusion specific mAb D25 and AM14 [18]. We reported that fewer ng of total anti-pre-F IgG in DS-Cav1 F, UC-2 F, or UC-3 F VLP sera compared to post-F VLP sera were required to block 50% binding of the pre-fusion specific mAbs. That is, the DS-Cav1 F, UC-2 F, and UC-3 F VLPs induced sera that had a higher concentration of D25-blocking and AM14-blocking antibodies/ng of total anti-pre-F binding IgG than the sera from post-F VLP immunization, as expected.

Using the same approach, we compared the specificities of the anti-pre-F IgG antibodies in the different sera from the pregnant CR dams by measuring the ng/mL of total anti-pre-F IgG that could block the binding of two different pre-fusion specific mAb, D25 and AM14. Appendix A, Figure A1, illustrates the approach for determination of the levels of binding of mAb with different dilutions of serum IgG. Then, the ng/mL of anti-pre-F IgG in the dilution of sera that resulted in 50% reduction in binding of the mAb could be determined. Conversion of the dilution of sera to ng/mL takes into account differences in the total anti-pre-F IgG in the different sera.

**Figure 1:**
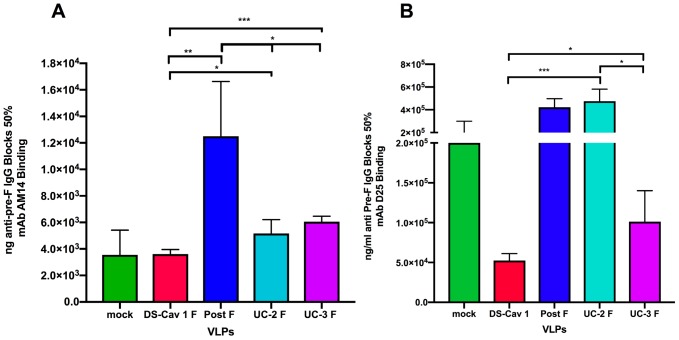
Blocking of binding of pre-fusion specific monoclonal antibodies (mAb) by sera from virus-like particle (VLP)-immunized dams. Shown are the concentrations (ng/mL) of total anti-pre-fusion F binding IgG that blocked 50% binding of mAb AM14 (panel A) or D25 (panel B) to soluble pre-fusion F protein (DS-Cav1) target in ELISA. Five groups of RSV primed, and pregnant CR dams were immunized with 100 μg of four different VLPs (DS-Cav1 F, Post F, UC-2 F, or UC-3 F VLPs) or mock at 2 weeks of gestation. Sera were harvested from each group at 4 weeks of gestation, just before the delivery of pups. Sera from each group were pooled, and the ng/mL of anti-pre-F binding IgG that blocked 50% of the binding of the mAb was determined as described previously [18] and in the Materials and Methods. Values above 4–5 × 10^5^ ng/mL in VLP sera indicate minimal or no blocking of the mAb. The results are the mean of three to five separate determinations with standard deviations indicated. *** *p* < 0.0005; ** *p* < 0.005; * *p* < 0.05.

Figure 1 shows ng/mL of anti-pre-F IgG in sera from groups of immunized dams that blocks AM14 (panel A) or D25 (panel B) binding to soluble pre-fusion F protein, DS-Cav1 F protein. Similar to murine sera, the concentration of AM14-blocking antibodies in DS-Cav1 F, UC-2 F, and UC-3 F VLP sera were similar, although the UC-2 and UC-3 F VLP sera had statistically significantly lower concentrations of AM14-blocking antibodies than the DS-Cav1 sera. DS-Cav1 F VLP sera had a somewhat higher concentration of D25-blocking antibodies compared to UC-3 F VLP sera (panel B). That is, more ng/mL of total anti-pre-F IgG in UC-3 F VLP sera were required to block D25 compared to DS-Cav1 F VLP sera. However, surprisingly, the UC-2 F VLP sera did not block D25 binding at any dilution of sera, similar to post-F VLP sera. The differences in levels of D25-blocking antibodies in sera induced by UC-2 F and UC-3 F VLPs may account, at least in part, for the previously reported higher NAb titers in dams and much better protection of pups after maternal immunization with UC-3 F VLPs compared to UC-2 F VLP immunization [30]. As a result of the apparent lack of D25-blocking antibodies in UC-2 F VLP sera, we proceeded to focus on comparisons of responses to DS-Cav1 F and UC-3 F VLPs.

As expected, the sera from post-F VLP immunizations weakly blocked AM14 binding compared to other sera, had no detectable levels of D25-blocking antibodies, and served as a negative control for these experiments.

The concentration of AM14 blocking anti-pre-F in sera from RSV primed, mock-immunized animals is similar to that of DS-Cav1 sera (Figure 1, panel A). However, it is clear that these sera have very low concentrations of D25-blocking antibodies in the total anti-pre-F IgG (Figure 1, panel B). This result may account, in part, to the previously reported low NAb titers in the sera of these animals and the poor protection of their offspring from RSV challenge.

### 3.2. Specificities of Anti-Pre-Fusion F Protein Antibodies Defined Using Soluble UC-3 F Targets

**Figure 2:**
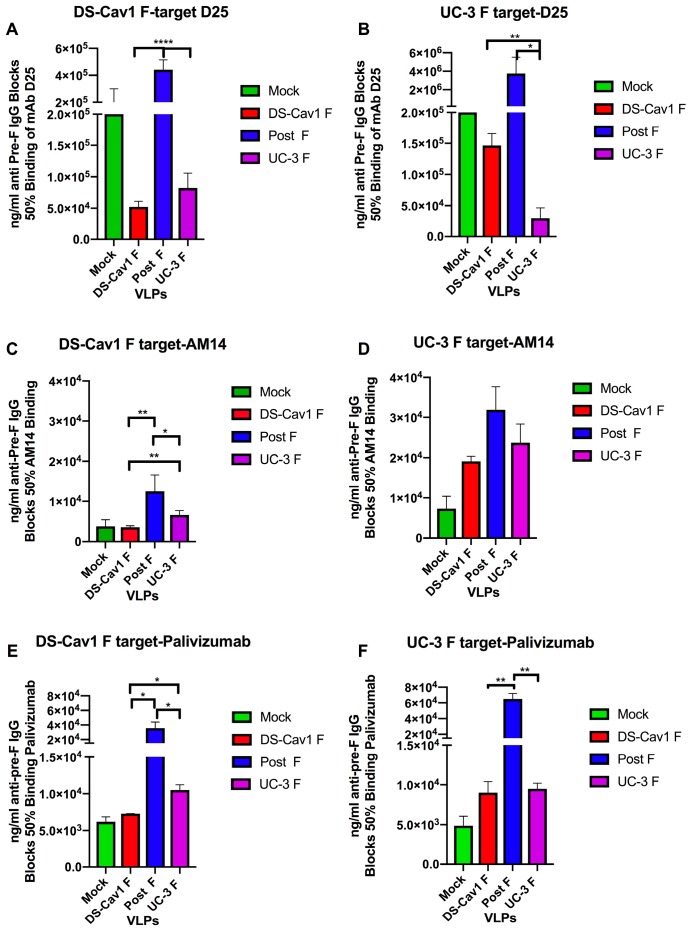
Blocking of binding of pre-fusion specific mAb by sera from VLP-immunized dams using different target antigens. Shown are the concentrations (ng/ml) of total anti-pre-fusion F binding IgG in VLP sera that blocked 50% binding of mAb D25 (panels A, B), AM14 (panels C, D), or palivizumab (panels E, F) to soluble DS-Cav1 F protein target (panels A, C, E) or soluble UC-3 F protein target (panels B, D, F) in ELISA. Determinations were done twice in parallel in order to accurately compare results with different targets. The results are the mean of the two separate determinations with standard deviations indicated. *** *p* < 0.0005; ** *p* < 0.005; * *p* < 0.05.

The results shown in Figure 1 were obtained using the soluble DS-Cav1 polypeptide as a target for serum antibody and mAb binding. The UC-3 F protein contains a single amino acid change in the region of the F protein identified as site ϕ (amino acids 61–76 and 195–210), which was the D25-binding site. While UC-3 F VLPs bind D25 at levels comparable to DS-Cav1 F VLPs [20,21], we considered the possibility that the differences in D25 blocking between UC-3 F sera and DS-Cav1 sera may be due to the altered site ϕ sequences in UC-3 F and thus altered populations of site ϕ antibodies induced by the different VLPs. Thus, we directly compared the results of D25 blocking with DS-Cav 1 F VLP or UC-3 F VLP sera, using a soluble DS-Cav1 F target (Figure 2, panel A) or soluble UC-3 F target (Figure 2, panel B). The post-F VLP sera serve as negative controls. The results showed that UC-3 F VLP sera could block D25 binding using either target. However, DS-Cav1 F VLP sera only very weakly blocked D25 binding to the UC-3 F target, while the UC-3 F VLP sera had a very high concentration of antibodies that blocked D25 binding to the UC-3 target. This result may indicate that UC-3 F VLP vaccination generates a broader spectrum of antibodies that are capable of recognizing alternate conformations of site ϕ [36,37].

As controls, we compared the blocking of mAb AM 14 (Figure 2, panels C and D) and palivizumab (Figure 2, panels E and F) binding to both targets using DS-Cav1 F VLP or UC-3 F VLP sera. Surprisingly, although the UC-3 F protein did not contain alterations in the binding sites of either mAb, relative levels of blocking of mAb AM14 binding to the two targets with DS-Cav1 F or UC-3 F VLPs sera were different. The concentrations of AM14-blocking antibodies or palivizumab-blocking antibodies in the two sera were the same using the UC-3 F target (Figure 2, panels D and F, respectively), but the concentrations of blocking antibodies measured with the DS-Cav1 target were statistically significantly different (Figure 2, panels C and E). These results suggest that the different mutations stabilizing the pre-fusion F protein affect the populations of antibodies they induce. These results may suggest that the increased protection afforded to pups by UC-3 F VLP maternal immunization compared to DS-Cav1 VLP immunization may be due, in part, to the induction of antibodies to site ϕ that can bind to a broader range of conformations of site ϕ.

### 3.3. Specificities of Anti-Pre-Fusion F Protein Antibodies in Offspring of Immunized Dams

Offspring of dams immunized with UC-3 F VLPs at 2 weeks of gestation were approximately 4-fold better protected from RSV challenge than the offspring of dams immunized with DS-Cav1 F VLPs [30] (Table 1). After RSV challenge, RSV lung titers in pups of the UC-3 F VLP-immunized dams were, on average, 4.7 × 10^2^ pfu/gm lung tissue, while the titers in lungs of pups from the DS-Cav1 F VLP-immunized dams were 1.8 × 10^3^ pfu/gm (Table 1), whereas the average titer of mock-vaccinated animals was 4.7 × 10^4^ pfu/gm. Since analyses of specificities of dam antibodies do not provide an unambiguous reason for better protection by UC-3 F VLP dam immunization, we characterized the specificities of populations of antibodies transferred from dams to pups, which was assessed as described above. Since we have previously reported that the antibody titers in all pups in the same litter were very similar [14], we pooled the sera of pups from the same litter. We assessed each pool separately to determine if there were differences in antibody transfer from different dams. Table 1 shows the average NAb titers of sera from individual pup pools as well as the average RSV titers in the lungs of each RSV-challenged pup in each pool.

**Figure 3:**
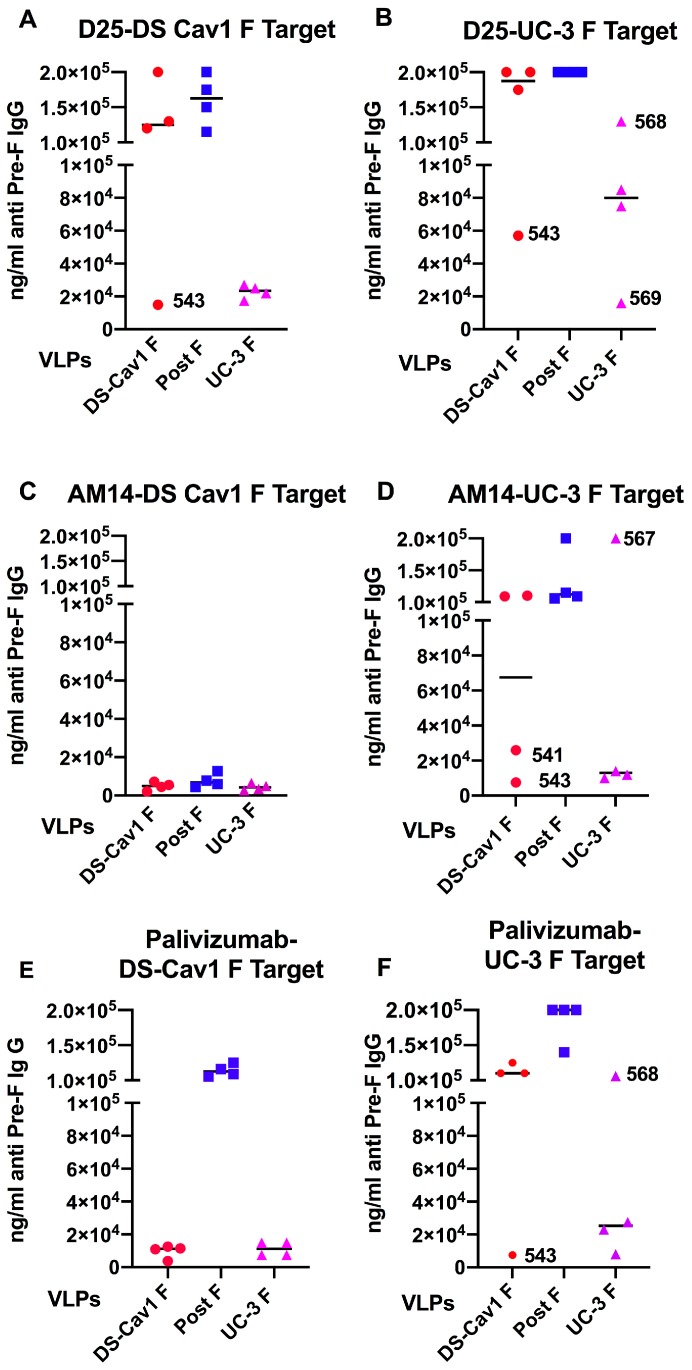
Blocking of binding of mAb by pup sera. The concentration (ng/ml) of anti-pre-F binding IgG required to block 50% binding of D25 (panels A, B), AM14 (panels C, D), or palivizumab (panels E, F) to soluble DS-Cav1 F (panels A, C, E) or UC-3 F protein (B, D, F) targets by ELISA. The sera of the offspring from a single dam were pooled, and each point is the average of two separate determinations of the concentrations of anti-pre-F IgG in each pool required to block 50% of the binding of each mAb. All pup sera pools contained 3 × 10^5^ ng/mL anti-pre-F binding IgG. Values above 100,000 ng/mL indicate very minimal or no blocking of binding of mAb. Numbers shown next to points identify the pup pools described in Table 1.

The blocking of mAb D25, AM14, or palivizumab by the sera from each pool was measured using either soluble targets DS-Cav1 F (Figure 3, panels A, C, E, respectively) or UC-3 F protein (Figure 3, panels B, D, F, respectively) for antibody binding. Shown are the ng/mL of total anti-pre-F IgG in each litter that blocks 50% of mAb D25 binding (Figure 3, panels A and B), 50% of mAb AM14 binding (Figure 3, panels C and D), or 50% of palivizumab binding (Figure 3, panels E and F). First, it was clear that the antibody populations in different litters of dams immunized with the same VLP can be quite different. For example, the D25-blocking antibodies were absent in three of the four litters of DS Cav1 F VLP-immunized animals. However, one of these litters (litter 543) had good levels of D25-blocking antibodies using the DS-Cav1 target and a 4 to 5-fold lower concentration using the UC-3 F target. In contrast, all four litters of UC-3 F VLP-immunized dams acquired D25-blocking antibodies measured using the DS-Cav1 target, although the levels were variable from litter to litter, particularly using the UC-3 target antigen. One of these litters (568) had no or low levels of D25-blocking antibodies using the UC-3 F target. These combined results, that the UC-3 F VLP immunization of dams results in a more consistent level of D25-blocking antibodies in pups compared to the DS-Cav1 F VLP immunization of dams, may account in part for the better protection, on average, of pups from RSV challenge after the UC-3 F VLP immunization of dams compared to DS-Cav1 F VLP immunization (Table 1).

It is significant that all litters had very high concentrations of AM14-blocking antibodies using the DS-Cav1 target (Figure 3, panel C), indicating that all pups had acquired maternal antibodies. Thus, a lack of D25-blocking antibodies in three litters of the DS-Cav1 F VLP-immunized animals cannot be due to a failure to transfer antibodies to pups. Further, all litters from dams immunized with either pre-fusion F VLP had high concentrations of antibodies that blocked the binding of palivizumab (Figure 3, panels E, F). However, there was variation between litters using the UC-3 F target for the binding of both AM14 and palivizumab, indicating that there is considerable variability in the transfer of these specific antibody populations from dams.

Thus, in summary, three-fourths of the litters from DS-Cav1 F VLP-immunized dams had virtually no detectable D25-blocking antibodies using either target, while all litters had high concentrations of AM14-like antibodies that blocked binding to the DS-Cav1 target. In contrast, all litters from UC-3 F VLP-immunized dams had antibodies that blocked D25 binding to the DS-Cav1 target, and all had antibodies that blocked AM14 binding to the soluble DS-Cav1 target. These combined results may indicate that the transfer of antibodies that can block D25 binding may account, in part, for the better protection of offspring by UC-3 F VLP immunization.

### 3.4. Levels of Anti-G Protein Antibodies in CR Sera

**Figure 4:**
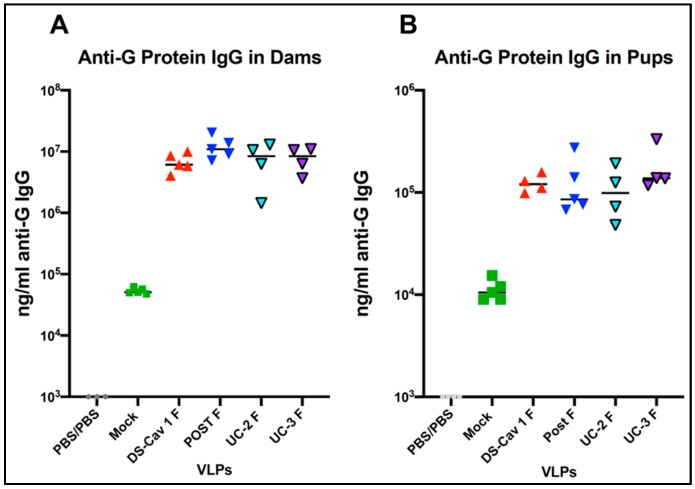
Induction of anti-G antibodies in VLP-immunized animals and their offspring. Five groups of RSV-primed, pregnant CR dams were immunized with 100 μg of four different VLPs (DS-Cav1 F, Post F, UC-2 F, or UC-3 F VLPs or mock immunized) at 2 weeks of gestation. Panel A shows the ng/mL of anti-G IgG from individual dams immunized at 2 weeks of gestation. Panel B shows the ng/mL of pooled pup sera from each dam. There were no significant differences between groups of dams or between groups of pups.

It has been shown that anti-G protein antibodies can provide protection from RSV challenge in animal models [38,39,40,41,42]. Thus, we considered the possibility that the differential protection of offspring of immunized dams could be due to the differential induction of anti-G antibodies by different VLPs. To address this question, we determined the levels of total anti-G protein antibodies in the serum of dams and in their offspring. Figure 4 shows levels of anti-G IgG in the sera of RSV primed dams two weeks after VLP immunization, (panel A), and in their offspring at 4 weeks after birth (panel B).

There are no significant differences between levels of anti-G IgG in dams or in levels in the pup sera, although the levels of antibodies in dams were approximately 100-fold higher than the levels in pups. Thus, the differences in the levels of total anti-G antibodies cannot account for the different responses to the different VLPs.

## 4. Discussion

The identification of multiple different mutation-stabilized pre-fusion F proteins led us to ask if these versions of pre-fusion F protein were indeed identical in terms of the conformation of the F proteins and, therefore, the properties of the immune responses upon their use as immunogens. We have addressed this question by comparing the representative mAb reactivity of five different VLP-associated pre-fusion F proteins and found significant differences in the VLP binding of these mAb, suggesting differences in their conformation [18]. We also showed that after the immunization of mice with five different pre-fusion F VLPs, two alternative pre-fusion F protein VLPs (UC-2 F VLPs and UC-3 F VLPs) induced 3 to 4-fold higher NAb titers than DS-Cav 1 F VLPs [18]. Using these two alternative pre-fusion F VLPs as immunogens in pregnant CRs, we showed that UC-3 F VLPs resulted in better protection of their offspring from RSV challenge compared to the offspring of dams immunized with DS-Cav1 F VLPs or UC-2 F VLPs [30].

To begin to understand the reasons for the increased pup protection after UC-3 F VLP immunization of their dams, we compared populations of antibodies induced in dams by the different VLPs and antibody populations transferred to their offspring. We quantified the concentrations of pre-fusion F specific serum antibodies in the different groups that blocked the binding of representative monoclonal antibodies to the target soluble pre-fusion F protein. We used mAb D25, a pre-fusion specific site ϕ antibody [20,21], and AM14, a trimer and pre-fusion specific antibody [43,44], as well as palivizumab, a site 2 antibody that binds both pre- and post-fusion F proteins [44].

The serum antibodies induced in dams by the three pre-fusion F VLPs (DS-Cav1 F, UC-2 F, and UC-3 F VLPs) all contained AM14-blocking antibodies, although the concentration of these antibodies was higher in serum after DS-Cav1 F VLP immunization compared to UC-2 F VLP or UC-3 F VLP immunization (Figure 1). The antibodies induced by DS-Cav1 F VLPs and UC-3 F VLPs in dams also blocked D25 binding, although the concentration of these antibodies was higher after DS-Cav1 F VLP immunization. Most striking was the absence of D25-blocking antibodies after UC-2 F VLP immunization. Since site ϕ, recognized by D25, is considered a dominant epitope for the induction of NAb, this result is consistent with the lower average NAb titers in the sera of dams immunized with UC-2 F VLPs compared to that in UC-3 F sera (Table 1). The UC-2 F protein varies from UC-3 F by a single amino acid change near the stalk at the base of the globular head domain of the pre-fusion F protein, which is a position that is very distant from site ϕ, the D25-binding site. However, alterations in this region of the molecule may affect the overall conformation of the protein used as an immunogen, affecting the D25-binding site.

The minor differences in the concentrations of D25 or AM14-blocking antibodies in dams after immunization with DS-Cav1 F VLPs and UC-3 F VLPs do not clearly account for the significantly increased protection from RSV challenge of offspring of UC-3 F VLP-immunized animals (Table 1). This issue led us to address the concentration of blocking antibodies using different target antigens for the blocking experiments (Figure 2). Comparing the blocking of mAb binding using soluble DS-Cav1 F or UC-3 F proteins as target antigens, we found that relative concentrations of blocking of mAb D25, AM14, or palivizumab by serum antibodies varied with the target antigen used. Most striking were the differences in the serum blocking of D25 binding to soluble DS-Cav1 F and UC-3 F target proteins. The UC-3 F VLPs induced antibodies that blocked D25 binding to both targets, while the DS-Cav1 F VLP induced sera that only very weakly blocked D25 binding to the UC-3 F target. The ability of the UC-3 F VLP sera to effectively block the binding to the two targets in contrast to DS-Cav1 F VLP sera may indicate that antibodies in the UC-3 F VLP sera more broadly recognize different conformations of site ϕ in the pre-fusion F proteins [36] and may contribute to the increased NAb in dams and the increased protection from RSV challenge of the offspring of these dams.

Assessment of antibodies transferred to the offspring of immunized dams yielded several surprising results (Figure 3), which likely further impact the protection levels of these pups from RSV challenge. Three of the pup litters from DS-Cav1 F VLP-immunized dams had no detectable D25-blocking antibodies using either target F protein, while all the pup litters from UC-3 F VLP-immunized dams had these antibodies, although their levels varied with the litter particularly assessed with the UC-3 F target antigen. All litters had high concentrations of AM14-blocking antibodies using the DS-Cav1 target and the pups from the pre-fusion F-immunized dams had high concentrations of palivizumab-blocking antibodies on the DS-Cav1 target. While there was more variability in the blocking of mAb AM14 and palivizumab using the UC-3 F target, these antibodies may contribute to the increased NAb titers and protection in the offspring of immunized dams compared to offspring from post F VLP-immunized dams (Table 1).

The polyclonal antibody blocking and binding of mAb to F protein targets may be due to several reasons, which are not mutually exclusive. The polyclonal sera may contain populations of antibodies specific for the binding site of the mAb, thus blocking the binding of that mAb. Alternatively, the polyclonal antibodies may prevent access of the mAb to its binding site by binding to sites surrounding the mAb-specific epitope. It is also possible that the polyclonal antibody binding of the target may alter the target protein’s conformation and thus the mAb binding site. These last two possibilities may account for the observation of the presence of mAb AM14-blocking antibodies in pup sera from dams immunized with post-F VLPs, although post-F VLPs do not bind this antibody [18]. A possible explanation is that post-F VLPs can boost antibodies that can obscure the AM14 binding site, leading to the inhibition of AM14 binding and the high concentrations anti-pre-F IgG in that sera that can block AM14 binding. The failure of the UC-2 F-induced antibodies in dams to block D25 binding or the variability of pup sera to block mAb binding to two different targets may be due to the different conformations of the target F or the induction of those changes by the binding of the polyclonal CR sera.

Another interesting finding is that antibodies with different specificities are differentially transferred from dams to their pups. Most striking is the lack of detection of D25-blocking antibodies in most pup litters of DS-Cav1 F VLP-immunized dams, even though their dams had robust levels of these antibodies. Thus, DS-Cav1 F VLPs did not fail to induce D25-blocking antibodies in dams. Furthermore, failure to transfer these D25-blocking antibodies to the offspring of DS-Cav1 F VLP-immunized animals cannot be due to a lack of transfer of total antibodies, since all these pups acquired high levels of AM14 and palivizumab-blocking antibodies. This surprising finding could be due to the different properties of the antibodies induced by the different forms of the F protein [45]. It has been reported that digalactosylated antibodies and NK cell-activating antibodies are selectively transferred across the human placenta [45]. Comparisons of the properties of antibodies induced in dams and those transferred to pups will be a topic of future studies.

A potential explanation for the differences in immune responses to different mutation stabilized pre-fusion F proteins may be the result of somewhat altered conformations of the pre-fusion protein due to the different mutations introduced. Another possibility is suggested by a recent analysis of the pre-fusion forms of HIV env [46,47,48], the influenza HA proteins [49], and the Ebola G protein [50]. Studies of fusion proteins have long hypothesized that the conformational change from pre- to post-fusion forms must involve multiple intermediates including several reversible pre-fusion conformations prior to conversion to irreversible intermediates on the path to the post-fusion form (for example, [51]). Recent studies have clearly shown these intermediates by various protocols including single-molecule FRET (Forester resonance energy transfer). Furthermore, Gilman et al. have reported recent results that are consistent with the detection of alternative conformations in the RSV pre-fusion F protein [36]. It has also been suggested that mutations introduced into the HIV env protein to stabilize the pre-fusion conformation do not stabilize env in its most native form but stabilize it in a conformational intermediate. Given these results with other viral fusion proteins as well as the RSV pre-fusion F protein, it is reasonable to suggest that different mutant pre-fusion F proteins are stabilized at different stages in the pathway to the post-fusion form, resulting in proteins with different conformations and different antigenicity.

## Figures and Tables

**Table 1:** Summary of Properties of Dam and Pup Sera.

Vaccine	Dam NAb Titers ^a^	Pup Litter ID Number (Litter Size)	Litter NAb Titers ^a^	Pup Lung Titers ^b^	D25-- DS-Cav1 Target ^c^	D25-- UC-3 F Target ^c^	AM14-- DS-Cav1 Target ^c^	AM14-- UC-3 F Target ^c^
DS Cav1 VLPs	10.88 10.42 7.14 8.54	541 (6) 542 (2) 543 (4) 544 (4)	5.09 7.99 5.17 5.35	2.6 × 10^3^ 1.0 × 10^2^ 4.2 × 10^3^ 1.3 × 10^2^	- - + -	- - +/- -	+ + + +	+ - + -
Post F VLPs	8.55 8.50 8.70 8.17 9.12	546 (7) 547 (4) 548 (5) 549 (4) 550 (3)	4.40 4.76 4.32 4.32 4.32	7.1 × 10^3^ 1.3 × 10^3^ 2.3 × 10^3^ 4.5 × 10^3^ 1.5 × 10^3^	- - - - -	- - - - -	+ + + + +	- - - - -
UC-2 F VLPs	8.94 8.96 10.23 10.26	555 (4) 556 (3) 557 (2) 558 (7)	4.86 4.50 4.39 4.32	2.0 × 10^2^ 1.9 × 10^3^ 2.6 × 10^3^ 4.5 × 10^3^	ND	ND	ND	ND
UC-3 F VLPs	10.37 10.57 10.46 10.32	567 (7) 568 (1) 569 (3) 570 (5)	5.26 6.15 7.44 7.28	3.3 × 10^2^ 1.2 × 10^2^ 1.0 × 10^2^ 2.4 × 10^2^	+ + + +	+/- - + +/-	+ + + +	- + + +

**^a^** NAb titers log 2; **^b^** lung RSV titers pfu/gm lung tissue; **^c^** summarizes data in Figure 3: +, competition with mAb binding; - no competition; +/-, weak competition; ND, not done.
